# The *Ala54Thr* Polymorphism of the Fatty Acid Binding Protein 2 Gene Modulates HDL Cholesterol in Mexican-Americans with Type 2 Diabetes

**DOI:** 10.3390/ijerph13010052

**Published:** 2015-12-23

**Authors:** Lorena M. Salto, Liming Bu, W. Lawrence Beeson, Anthony Firek, Zaida Cordero-MacIntyre, Marino De Leon

**Affiliations:** 1 Center for Health Disparities and Molecular Medicine, Department of Basic Sciences, School of Medicine, Loma Linda University, Loma Linda, CA 92350, USA; lsalto@llu.edu (L.M.S.); buliming2002@yahoo.com (L.B.); lbeeson@llu.edu (W.L.B.); zcordero-macintyre@llu.edu (Z.C.-M.); 2 Center for Nutrition, Healthy Lifestyle, and Disease Prevention, School of Public Health, Loma Linda University, Loma Linda, CA 92350, USA; 3 Endocrinology Section, JL Pettis Memorial VA Medical Center, Loma Linda, CA 92357, USA; anthony.firek@va.gov

**Keywords:** FABP2, *Ala54Thr* polymorphism, Mexican-Americans, type 2 diabetes interventions

## Abstract

The alanine to threonine amino acid substitution at codon 54 (*Ala54Thr*) of the intestinal fatty acid binding protein (FABP2) has been associated with elevated levels of insulin and blood glucose as well as with dyslipidemia. The aim of this study was to characterize the effect of this FABP2 polymorphism in Mexican-Americans with type 2 diabetes (T2D) in the context of a three-month intervention to determine if the polymorphism differentially modulates selected clinical outcomes. For this study, we genotyped 43 participant samples and performed *post-hoc* outcome analysis of the profile changes in fasting blood glucose, HbA1c, insulin, lipid panel and body composition, stratified by the *Ala54Thr* polymorphism. Our results show that the Thr54 allele carriers (those who were heterozygous or homozygous for the threonine-encoding allele) had lower HDL cholesterol and higher triglyceride levels at baseline compared to the Ala54 homozygotes (those who were homozygous for the alanine-encoding allele). Both groups made clinically important improvements in lipid profiles and glycemic control as a response to the intervention. Whereas the Ala54 homozygotes decreased HDL cholesterol in the context of an overall total cholesterol decrease, Thr54 allele carriers increased HDL cholesterol as part of an overall total cholesterol decrease. We conclude that the *Ala54Thr* polymorphism of FABP2 modulates HDL cholesterol in Mexican-Americans with T2D and that Thr54 allele carriers may be responsive in interventions that include dietary changes.

## 1. Introduction

Type 2 diabetes (T2D) disproportionately affects Mexican-Americans in the United States [1]. Although genetic predisposition is a widely accepted risk factor for T2D, there has been modest progress in identifying and characterizing the specific genes that can contribute to this disease in Mexican-Americans. The intestinal fatty acid binding protein (FABP2) gene, and its respective alanine to threonine polymorphism at codon 54 (*Ala54Thr*), was first implicated as a likely candidate gene for insulin resistance in the Pima Indian population [2]. Genetic linkage analysis in non-diabetic Mexican-Americans provided further evidence for a proposed FABP2 gene role in insulin dysfunction [3]. Since then, the common *Ala54Thr* missense polymorphism has been associated with T2D in Chilean elders [4] and in Mexican-Americans [5], but not in African-Americans [6] or Tongan Polynesians [7]. In a meta-analysis study, the *Ala54Thr* polymorphism was weakly associated with higher insulin levels and higher fasting blood glucose levels in some but not all ethnic populations [8]. Ethnic-specific associations between the *Ala54Thr* polymorphism and risk of T2D are also supported by other meta-analysis publications [9,10]. The association between the *Ala54Thr* polymorphism and dyslipidemia overall seems to be stronger [11]. For example, several clinical studies on Mexican patients report elevated triglyceride levels in FABP2 Thr54 allele carriers (Ala/Thr and Thr/Thr, those who are heterozygous or homozygous for the threonine-encoding allele) [12,13,14]. Other studies on U.S. populations have only reported on African Americans and non-Hispanic Whites, with conflicting ethnic and gender specific results [6,15,16,17].

The FABP2 gene encodes the intestinal fatty acid binding protein, a member of the 14–15 kDa intracellular lipid binding family of proteins. Specifically, FABP2 is expressed in enterocytes and preferentially binds saturated and unsaturated long-chain fatty acids. A single nucleotide substitution from G to A at codon 54 of exon 2 results in the missense mutation that gives rise to the alanine-to-threonine (*Ala54Thr*) amino acid substitution in the coded protein [2]. *In-vitro* evidence shows that the threonine-encoded protein results in a twofold greater fatty acid binding affinity [2] and is associated with an increase in fat absorption in explants [18] and in T2D patients [19]. Due to the inherent role of FABP2 in the absorption and transport of long-chain fatty acids within enterocytes, the increased binding affinity associated with the Thr54 variant of the *Ala54Thr* polymorphism may give rise to a genetic and environmental interaction. For instance, high fat intake, coupled with higher lipid oxidation rates, may induce insulin and glucose dysfunction in non-diabetic Thr54 allele carriers [20,21]. Previous studies have shown that FABP2 Thr54 allele carriers are susceptible to dietary fat; those studies have also typically reported adverse outcomes for high saturated fat intake levels [17,19,20,22,23]. Thr54 allele carriers often show an increased risk for insulin resistance in the context of a high saturated fat diet but may show positive outcomes in the context of a high omega-3 polyunsaturated fat diet [17,24,25]. The evidence suggests that the burden of this FABP2 polymorphism may be subject to lifestyle factors. However, it is still unclear whether this polymorphism can play a role in modulating T2D clinical outcomes in an educational intervention that advocates lifestyle changes. This study determined whether the *Ala54Thr* polymorphism of FABP2 affected the blood glucose and lipid parameters of Mexican-Americans with type 2 diabetes using data from a diabetes education program. Specifically, this study assessed whether the *Ala54Thr* polymorphism of FABP2 modulated changes in glycemic control, serum lipid profiles and body composition values for participants of a three-month diabetes education intervention.

## 2. Experimental Section

### 2.1. Methods

We report on data collected for the *En Balance* diabetes education program at Loma Linda University. Briefly, *En Balance* is a hands-on, culturally-competent, three-month diabetes education intervention for Spanish-speaking Hispanics with T2D [26]. The *En Balance* study was approved by the Loma Linda University Institutional Review Board. The intervention objectives were to improve glycemic control, change dietary habits and increase physical activity in underserved San Bernardino and Riverside county Mexican-Americans. The participants of this intervention were recruited through posted flyers and through community clinic physician referrals. Those that agreed to participate and also signed an informed consent attended a baseline data collection clinic that included food-frequency and physical activity questionnaire interviews, baseline body composition measurements and the collection of fasting blood samples. After the baseline data collection, the participants attended the comprehensive, culturally-tailored diabetes education program [26]. The education component consisted of four, two-hour long presentations that taught the participants how to manage their diabetes, improve their dietary intake and increase their physical activity. A trained nutrition professional presented the lessons using a hands-on, culturally relevant approach that focused on smaller portion sizes and healthier alternatives within culturally specific foods. Once the participants completed the educational component of the program and after three months from baseline had elapsed, they attended a three-month data collection clinic to repeat all of the baseline measures. For this secondary data analysis, we evaluated genotype and paired clinical lab data from blood samples collected for 43 participants; of those, eleven were males and thirty-two were females. We analyzed genotype, fasting glucose, HbA1c, insulin, lipid profiles (HDL, LDL, total cholesterol and triglycerides), anthropometric measurements, and body composition values for each participant at baseline and at three-months. We used bioelectrical impedance analysis (BIA) and dual-energy X-ray absorptiometry (DXA) (Hologic Fan Beam, Discovery A Software Version) methods to measure body composition. We determined the genotype data for the *En Balance* samples using PCR (PCR-CTPP), the TaqMan Allelic Discrimination Assay and the Sequence Detection Systems (SDS) v2.3 software (Applied Biosystems, Thermo Fisher Scientific Inc., Grand Island, NY, USA).

### 2.2. Statistical Analysis

We performed all of the statistical analyses with SPSS Statistics Software version 22.0 (IBM: SPSS, Armonk, New York, NY, USA). We compared the Ala54 homozygotes (those with a G/G genotype and therefore homozygous for the alanine-encoding allele) to the Thr54 allele carriers (those with G/A or A/A genotypes and therefore heterozygous or homozygous for the threonine-encoding allele). All of the genotype frequencies were in agreement with Hardy-Weinberg equilibrium expectations as tested by the chi-square goodness-of-fit statistic. To test both the within-subjects factor (the paired-sample means at baseline and three-months) and the between-subjects factor (the *Ala54Thr* polymorphism subgroup) we ran a mixed General Linear Model. We also used independent samples *t*-tests, Mann-Whitney U tests, paired-samples *t*-tests, and Wilcoxon signed-ranks to compare the baseline and three-month means stratified by the *Ala54Thr* polymorphism subgroups and also for the combined group. To determine whether the baseline value of each outcome variable, in it of itself, affected the change seen at three-months in that same variable of interest, we ran a linear regression model for each with the change variable (three-month minus baseline) as the dependent variable and the baseline variable as the primary independent variable adjusted for the effect of the *Ala54Thr* polymorphism. We tested whether BMI, age, or gender modified the observed outcome variables at baseline by including the *Ala54Thr* polymorphism along with each demographic variable on its own and the corresponding interaction variable (BMI, age or gender and *Ala54Thr* polymorphism product term) in linear regression equations. We corrected those results for multiple comparisons using the Bonferroni correction. The HOMA-IR equation [27,28] was used to estimate insulin resistance and the type 1 error was set at α = 0.05 for statistical significance. The data are shown as mean ± SD, except where noted.

## 3. Results

Table 1 shows the FABP2 *Ala54Thr* polymorphism genotype and allele frequencies for the *En Balance* Mexican-American adults with type 2 diabetes. The proportion of Mexican-American Thr54 allele carriers ranged from 55% in the males to 63% in the females, or 61% when combined. Proportionally, females were more likely to be Thr54 allele carriers than males, although this difference was not statistically significant.

The *En Balance* study participants ranged from 29 to 75 years of age and had a group mean and median age of 53.1 ± 10.4 (SD) and 52 years, respectively. The characteristics for the complete participant group at baseline are summarized in Table 2 according to the FABP2 *Ala54Thr* polymorphism subgroups. Males who were Thr54 allele carriers were significantly older (*p* = 0.04) than their Ala54 homozygous counterparts but no significant age difference was seen in the female subgroup or in the age, weight, or body mass index of the Ala54 homozygous group when compared to the Thr54 allele carrier group (See Table 2). At baseline, age, gender and BMI did not modify the observed outcome variables when each was tested for effect modification and after correcting for multiple comparisons. The Ala54 homozygous group means were comparable to the Thr54 allele carrier group means for all baseline measures except for HDL cholesterol and triglycerides as tested by independent samples *t*-tests or Mann-Whitney U tests (Table 2). The Thr54 allele carriers had higher triglyceride levels (*p* = 0.036) and lower HDL cholesterol levels (*p* = 0.030) when compared to the Ala54 homozygotes; Thr54 allele carriers also demonstrated higher fasting blood glucose levels at baseline, although this difference was not statistically significant (*p* = 0.269).

**Table 1:** *En Balance* Mexican-American FABP2 *Ala54Thr* polymorphism ^a^ genotype and allele frequencies.

*Ala54Thr* Genotype ^b^ /Polymorphism	G/G (Ala/Ala)		G/A (Ala/Thr)		A/A (Thr/Thr)		Thr54 Allele Carrier		Allele Frequency	
	*n*	%	*n*	%	*n*	%	*n*	%	Ala54	Thr54
*En Balance* Mexican-Americans										
All	17	39.5%	18	41.9%	8	18.6%	26	60.5%	0.60	0.40
Males	5	45.5%	3	27.3%	3	27.3%	6	54.5%	0.59	0.41
Females	12	37.5%	15	46.9%	5	15.6%	20	62.5%	0.61	0.39

^a^ Ala/Ala (Ala54 homozygotes, G/G genotype) *vs.* Ala/Thr and Thr/Thr (Thr54 allele carriers, G/A and A/A genotypes combined); ^b^ All of the genotype frequencies were in agreement with Hardy-Weinberg equilibrium expectations as tested by the chi-square goodness-of-fit statistic.

**Table 2:** Baseline blood glucose, lipid and body composition profiles for the *En Balance* participants stratified by the FABP2 *Ala54Thr* polymorphism ^a^.

Polymorphism	Ala/Ala		Ala/Thr and Thr/Thr		Mean Difference	*p*-Value ^b^
	*n*	Mean ± SD	*n*	Mean ± SD	± Standard Error	
Age (years)	17	54.0 ± 10.6	26	52.6 ± 10.5	1.34 ± 3.31	0.654
Fasting Blood Glucose (mg/dl)	17	148.5 ± 74.4	26	171.6 ± 72.8	23.1 ± 22.9	0.269
HbA1c (%)	17	8.1 ± 2.6	26	8.1 ± 2.3	0.03 ± 0.78	0.804
Insulin (uU/mL)	17	15.1 ± 10.2	26	13.3 ± 9.7	1.78 ± 3.10	0.611
HOMA-IR ^c^	15	4.39 ± 2.3	25	5.13 ± 3.1	0.73 ± 0.94	0.459
Total Cholesterol (mg/dl)	17	191.7 ± 41.0	26	184.6 ± 41.1	7.01 ± 12.82	0.628
HDL Cholesterol (mg/dl)	17	51.0 ± 12.5	26	42.8 ± 7.1	8.25 ± 3.01	0.030 *
LDL Cholesterol (mg/dl)	17	118.5 ± 37.4	26	114.0 ± 31.0	4.51 ± 10.51	0.785
Cholesterol: HDL ratio	17	3.9 ± 1.1	26	4.3 ± 1.0	0.45 ± 0.34	0.132
Triglycerides (mg/dl)	17	187.3 ± 159.6	26	220.8 ± 90.1	33.4 ± 38.1	0.036 *
BIA: Fat Mass (kg)	16	31.8 ± 12.4	24	34.9 ± 13.9	3.01 ± 4.31	0.590
BIA: Fat %	16	37.8 ± 10.4	24	41.0 ± 8.3	3.15 ± 2.98	0.362
BIA: Fat-free Mass (kg)	16	48.7 ± 10.5	23	47.8 ± 8.7	0.88 ± 3.10	0.932
Weight (kg)	16	80.0 ± 18.3	26	82.0 ± 18.5	2.09 ± 5.87	0.806
Waist Circumference (cm)	17	99.3 ± 13.6	26	101.0 ± 13.5	1.73 ± 4.23	0.593
Hip Circumference (cm)	17	110.5 ± 15.3	26	110.9 ± 16.0	0.48 ± 4.92	0.794
Waist to Hip Ratio	17	0.90 ± 0.06	26	0.91 ± 0.06	0.01 ± 0.02	0.747
BMI (kg/m^2^)	17	32.1 ± 6.7	26	32.6 ± 7.3	0.54 ± 2.27	0.907
DXA: Trunk Fat (kg)	17	15.9 ± 6.7	26	16.4 ± 6.4	0.55 ± 2.05	0.785
DXA: Trunk Lean (kg)	17	25.2 ± 4.9	26	25.6 ± 4.8	0.36 ± 1.52	0.980
DXA: Trunk Percent Fat (%)	17	37.0 ± 9.7	26	37.6 ± 8.6	0.63 ± 2.83	0.941
DXA: Total Fat (kg)	17	30.2 ± 11.4	26	31.5 ± 13.1	1.39 ± 3.90	0.882
DXA: Total Lean (kg)	17	49.0 ± 10.3	26	49.2 ± 9.7	0.15 ± 3.11	0.960
DXA: Total Percent Fat (%)	17	36.5 ± 9.3	26	37.1 ± 8.9	0.60 ± 2.84	0.891

BIA = bioelectrical impedance analysis; BMI = body mass index; DXA = dual-energy X-ray absorptiometry; ^a^ Ala/Ala (Ala54 homozygotes, G/G genotype) *vs.* Ala/Thr and Thr/Thr (Thr54 allele carriers, G/A and A/A genotypes combined); ^b^ This *p*-value is based on independent samples *t*-tests or Mann-Whitney U tests comparing the *Ala54Thr* polymorphism subgroups; * *p*-value < 0.05; ^c^ Homeostasis Model Assessment of Insulin Resistance (HOMA-IR) = [fasting insulin (mU/L) × fasting glucose (mmol/L)] / 22.5.

**Table 3:** Baseline to three-month *En Balance* participant changes stratified by the FABP2 *Ala54Thr* polymorphism ^a^.

Polymorphism	Ala/Ala (*n* = 17)			Ala/Thr and Thr/Thr (*n* = 26)			Within ^b^ Subjects *p*-Value	Between ^c^ Subjects *p*-Value
	Baseline	Three-Month	Mean Difference	Baseline	Three-Month	Mean Difference		
	Mean ± SD	Mean ± SD	± SD	Mean ± SD	Mean ± SD	± SD		
FBG (mg/dl)	148.5 ± 74.4	133.9 ± 47.6	−14.5 ± 60.3	171.6 ± 72.8	149.0 ± 65.2	−22.69 ± 34.7	0.014 *	0.332
HbA1c (%)	8.1 ± 2.6	7.0 ± 1.2	−1.03 ± 1.91	8.1 ± 2.3	7.5 ± 2.0	−0.64 ± 1.18	0.001 *	0.714
Insulin (uU/mL)	15.1 ± 10.2	15.4 ± 9.5	0.34 ± 7.12	13.3 ± 9.7	15.0 ± 14.6	1.64 ± 7.59	0.395	0.742
HOMA-IR ^d^	4.39 ± 2.3	4.28 ± 2.5	−0.11 ± 2.85	5.13 ± 3.1	4.28 ± 2.4	−0.84 ± 1.83	0.203	0.649
Total Cholesterol (mg/dl)	191.7 ± 41.0	178.8 ± 50.4	−12.8 ± 30.4	184.6 ± 41.1	174.6 ± 50.2	−10.0 ± 37.5	0.042 *	0.674
HDL Cholesterol (mg/dl)	51.0 ± 12.5	49.0 ± 12.3	−2.00 ± 7.90	42.8 ± 7.1	44.2 ± 8.2	1.42 ± 5.84	0.785	0.029 *
LDL Cholesterol (mg/dl)	118.5 ± 37.4	108.5 ± 39.1	−10.0 ± 29.4	114.0 ± 31.0	105.8 ± 34.9	−8.19 ± 27.2	0.045 *	0.722
Cholesterol: HDL ratio	3.9 ± 1.1	3.8 ± 1.2	−0.12 ± 0.83	4.3 ± 1.0	4.0 ± 1.0	−0.36 ± 0.69	0.041 *	0.310
Triglycerides (mg/dl)	187.3 ± 159.6	172.7 ± 114.3	−14.5 ± 86.3	220.8 ± 90.1	200.9 ± 121.6	−19.8 ± 82.2	0.195	0.387
BIA: Fat Mass (kg)	31.8 ± 12.4	29.9 ± 12.1	−1.97 ± 4.41	34.9 ± 13.9	33.3 ± 13.8	−1.54 ± 1.93	0.001 *	0.453
BIA: Fat %	37.8 ± 10.4	36.6 ± 9.8	−1.24 ± 3.11	41.0 ± 8.3	39.4 ± 8.7	−1.57 ± 1.73	0.001 *	0.317
BIA: Fat-free Mass (kg)	48.7 ± 10.5	49.2 ± 10.6	0.52 ± 1.91	47.8 ± 8.7	48.4 ± 8.7	0.60 ± 1.51	0.046 *	0.787
Weight (kg)	80.0 ± 18.3	79.6 ± 17.2	−0.39 ± 3.21	82.0 ± 18.5	81.6 ± 18.5	−0.41 ± 2.19	0.339	0.720
Waist Circumference (cm)	99.3 ± 13.6	97.9 ± 11.5	−1.34 ± 5.75	101.0 ± 13.5	99.2 ± 14.4	−1.86 ± 4.98	0.059	0.721
Hip Circumference (cm)	110.5 ± 15.3	110.8 ± 13.0	0.31 ± 4.05	110.9 ± 16.0	110.4 ± 15.6	−0.53 ± 2.85	0.829	0.989
Waist to Hip ratio	0.90 ± 0.06	0.88 ± 0.04	−0.02 ± 0.06	0.91 ± 0.06	0.90 ± 0.06	−0.01 ± 0.05	0.072	0.454
BMI (kg/m^2^)	32.1 ± 6.7	31.7 ± 6.1	−0.39 ± 1.32	32.6 ± 7.3	32.2 ± 7.3	−0.44 ± 0.85	0.017 *	0.817
DXA: Trunk Fat (kg)	15.9 ± 6.7	15.5 ± 5.8	−0.36 ± 1.42	16.4 ± 6.4	16.0 ± 6.6	−0.43 ± 0.74	0.021 *	0.798
DXA: Trunk Lean (kg)	25.2 ± 4.9	25.3 ± 5.2	0.06 ± 1.06	25.6 ± 4.8	25.5 ± 4.7	−0.08 ± 1.04	0.962	0.849
DXA: Trunk Percent Fat (%)	37.0 ± 9.7	36.8 ± 8.9	−0.20 ± 2.12	37.6 ± 8.6	36.9 ± 8.8	−0.73 ± 1.41	0.089	0.895
DXA: Total Fat (kg)	30.2 ± 11.4	29.5 ± 10.6	−0.62 ± 1.97	31.5 ± 13.1	30.9 ± 13.0	−0.64 ± 1.37	0.017 *	0.721
DXA: Total Lean (kg)	49.0 ± 10.3	49.2 ± 10.8	−0.15 ± 1.89	49.2 ± 9.7	49.2 ± 10.0	0.02 ± 1.40	0.786	0.983
DXA: Total Percent Fat (%)	36.5 ± 9.3	36.1 ± 9.2	−0.31 ± 1.35	37.1 ± 8.9	36.5 ± 9.0	−0.53 ± 1.09	0.028 *	0.863

FBG = Fasting blood glucose; BIA = bioelectrical impedance analysis; BMI = body mass index; DXA = dual-energy X-ray absorptiometry; ^a^ Ala/Ala (Ala54 homozygotes, G/G genotype) *vs.* Ala/Thr and Thr/Thr (Thr54 allele carriers, G/A and A/A genotypes combined); ^b^ This p-value is based on the within-subjects factor results of the mixed General Linear Model analysis comparing baseline and three-month means; * *p*-value < 0.05; ^c^ This p-value is based on the between-subjects factor results of the mixed General Linear Model analysis comparing the *Ala54Thr* polymorphism subgroups; * *p*-value < 0.05; ^d^ Homeostasis Model Assessment of Insulin Resistance (HOMA-IR) = [fasting insulin (mU/L) × fasting glucose (mmol/L)] / 22.5, *n* = 40 for HOMA-IR.

As a combined group, the participants made clinically important decreases in glycemic control, serum lipid profiles and body composition parameters at the end of the three-month diabetes education intervention (see Supplementary Table S1). Both *Ala54Thr* polymorphism subgroups made clinically and statistically significant decreases in fasting blood glucose and HbA1c, as well as in total cholesterol, LDL cholesterol and cholesterol/HDL ratio (see Table 3). Both subgroups also made small, but statistically significant changes in BIA fat mass, BIA fat percent, BIA fat-free mass, BMI, DXA trunk fat, DXA total fat and DXA total percent fat from baseline to three-months (Table 3). Within the context of an overall total cholesterol decrease, the Ala54 homozygotes and the Thr54 allele carriers decreased LDL cholesterol and triglycerides; however, the Thr54 allele carriers increased HDL cholesterol whereas the Ala54 homozygotes decreased HDL cholesterol at three-months. This difference in subgroup response was statistically significant (*p* = 0.029) even though the mean differences from baseline to three-months were not (See Table 3). When we tested the baseline value of each measured outcome to see if the baseline level alone influenced the change seen at three-months, we found that the baseline levels of fasting blood glucose, HbA1c, and LDL cholesterol alone were significantly correlated to their statistically significant decreases at three-months (see Supplementary Table S2). The baseline levels of total cholesterol and HDL cholesterol on their own, however, were not significantly predictive of their three-month changes.

## 4. Discussion

We previously reported an association between the Thr54 variant of FABP2 and type 2 diabetes in Mexican-Americans [5]. This current study expands those findings by first characterizing the fasting blood glucose, insulin, total cholesterol, HDL cholesterol, LDL cholesterol, and triglyceride values in Mexican-Americans with type 2 diabetes using samples from our *En Balance* study. Our findings suggest that the *Ala54Thr* polymorphism plays a distinct role in the lipid and glucose diabetes risk profiles of Mexican-Americans. In this study, approximately 61% of the diabetes education intervention participants were Thr54 allele carriers and the Thr54 allele frequency (0.395) was higher than what has been reported for other US ethnic groups (Pima Indians with T2D: 0.28, Non-Hispanic Whites with T2D: 0.27, Blacks with T2D: 0.22) [2,6,15]. Our results *at baseline* are consistent with the view of the *Ala54Thr* polymorphism as a risk-factor: the Thr54 allele carriers had higher triglyceride and lower HDL cholesterol levels when compared to the Ala54 homozygotes in an age-comparable, weight-comparable and body composition-comparable Mexican-American group with T2D [12,13,14,16,18,19,29]. We also observed higher fasting blood glucose and insulin resistance levels in the Thr54 allele carriers at baseline although these mean differences were not statistically significant relative to the Ala54 homozygotes [2,17,21,22,23,29,30,31].

Although the Thr54 allele carriers demonstrated worse HDL cholesterol and triglyceride profiles than their Ala54 homozygous counterparts at baseline, our data suggest that they also made comparable clinical improvements in glycemic control, serum lipid profiles and body composition values from baseline to three-months. We did not find that Thr54 allele carriers were more resistant to changes [32,33]; rather, with regard to HDL cholesterol, we found that the Thr54 allele carriers demonstrated a better response than the Ala54 homozygotes, even while both groups decreased LDL and total cholesterol. While we could not test the effect of diet due to incomplete food frequency questionnaire data, we suspect that dietary fat intake may be an influential factor. One of the *En Balance* intervention objectives was to promote healthy dietary intake changes in the participants and we have previously reported on the positive dietary fat and cholesterol intake decreases as a result of participation in the intervention [26].

Our data are consistent with other studies that report a non-deleterious role for the Thr54 variant of the *Ala54Thr* polymorphism in an intervention [34,35] and are in line with studies where the Thr54 allele carriers experienced greater decreases in total cholesterol, LDL cholesterol and apolipoprotein B with changes in dietary intake [36,37]. Additionally, two studies have characterized the Thr54 variant as a factor that may give rise to beneficial outcomes in conjunction with omega-3 polyunsaturated fatty acid intake. In Keewatin Inuit, lower glucose concentrations were reported for Thr54 allele carriers instead of Ala54 homozygotes; an effect likely due to the increased binding affinity assigned to the Thr54 variant for omega-3 fatty acids [24]. The Thr54 variant also modulated HDL cholesterol in an eicosapentaenoic acid (EPA) supplementation study where the Thr54 allele carriers made greater increases in HDL when compared to the Ala54 homozygotes [25].

Given the role of FABP2 as an intracellular transporter of long-chain fatty acids in the small intestine, the clinical manifestation of the *Ala54Thr* polymorphism may be best understood by taking into account fatty acid intake. Baier *et al.* first suggested the possibility that the severity of this polymorphism would depend on the type of dietary fat [2] and as such, the studies that followed tested the differential effect of fatty acids on carriers and non-carriers of the Thr54 variant [17,19,21,22,23]. According to the hypothesized role of the Thr54 variant, insulin resistance, blood glucose and blood lipid profiles are aggravated in Thr54 allele carriers due to the increased binding affinity for saturated fatty acids or due to the absorption of saturated fatty acids at a more rapid rate [2,17,18,21,34]. The altered intracellular binding affinity attributed to the Thr54 variant may improve insulin, blood glucose, blood lipid, and body composition parameters in Thr54 allele carriers who consume high amounts of monounsaturated or polyunsaturated fats [24,25,37,38]. This differential modulation may help reconcile conflicting reports regarding the associations between the *Ala54Thr* polymorphism and insulin resistance, blood glucose, lipid profiles and type 2 diabetes in different ethnic groups [4,5,6,7,8,9,10,15,16]. When susceptibility to dietary fat and to changes in type of dietary fat are accounted for, it follows that the blood glucose and lipid profiles of Thr54 allele carriers would change more in response to an intervention [25,31,34,35]. While the published literature supports this inference, the underlying mechanism that specifically drives genetic predisposition, dietary fat intake and metabolism in Thr54 allele carriers is unknown.

This study has several limitations. We report *post-hoc* outcome analyses on a study with a small sample size that is further reduced by comparing the groups according to the *Ala54Thr* polymorphism of FABP2. Although in this sample the number of Thr54 allele carriers (*n* = 26) was greater than the number of Ala54 homozygotes (*n* = 17), the comparison between the two groups does not exceed the recommended 1:1.5 sample size ratio required for a balanced analysis. While these data show statistical significance in the participants of our intervention, they also provide the basis for additional T2D clinical interventions using larger sample sizes and a longer follow-up to fully understand if dietary intake and compliance may be involved.

## 5. Conclusions

Mexican-Americans in the U.S. are experiencing westernization trends in their traditional diet and lifestyle [26] that are changing with the increasing obesity and T2D prevalence rates [1]. Arguably, a high-saturated fat diet may burden Mexican-American Thr54 allele carriers to a greater degree than Mexican-American Ala54 homozygotes even when age and BMI are comparable between the two groups. Knowledge of this genetic and dietary interaction presents unique intervention opportunities that may decrease the health disparities seen in this population where roughly 54%–61% are Thr54 allele carriers.

Our findings support our hypothesis that the *Ala54Thr* polymorphism of FABP2 plays a distinct role in Mexican-Americans with type 2 diabetes. Specifically, the *Ala54Thr* polymorphism of FABP2 modulates HDL cholesterol in Mexican-Americans with type 2 diabetes; our results also indicate that Thr54 allele carriers may be responsive in interventions that promote changes in dietary fat intake.

## Supplementary Files

Supplementary File 1
