# Supplementary material for: The Ala54Thr Polymorphism of the Fatty Acid Binding Protein 2 Gene Modulates HDL Cholesterol in Mexican-Americans with Type 2 Diabetes
Source: Int J Environ Res Public Health. 2015 Dec 23;13(1):52. doi: 10.3390/ijerph13010052 (PMC4730443; doi:10.3390/ijerph13010052)
Supplement: Supplementary File 1 [file ijerph-13-00052-s001.pdf]

Supplementary Materials: The *Ala54Thr* Polymorphism of the Fatty Acid Binding Protein 2 Gene Modulates HDL Cholesterol in Mexican-Americans with Type 2 Diabetes

Lorena M. Salto, Liming Bu, W. Lawrence Beeson, Anthony Firek, Zaida Cordero-MacIntyre and Marino De Leon

Table S1. Baseline to three-month *En Balance* participant changes for the combined group (n = 43).

	Baseline	Three-Month	Mean Difference	*p*-Value [a]
	Mean ± SD	Mean ± SD	± SD	
Fasting Blood Glucose (mg/dl)	162.5 ± 73.4	143.1 ± 58.8	19.5 ± 46.1	0.008 *
HbA1c (%)	8.1 ± 2.5	7.3 ± 1.8	0.79 ± 1.51	0.001 *
Insulin (uU/mL)	14.1 ± 9.9	15.2 ± 12.7	1.13 ± 7.35	0.319
HOMA-IR [b]	4.85 ± 2.86	4.29 ± 2.49	0.57 ± 2.26	0.119
Total Cholesterol (mg/dl)	187.5 ± 40.8	176.3 ± 49.8	11.1 ± 34.6	0.041 *
HDL Cholesterol (mg/dl)	46.1 ± 10.4	46.1 ± 10.2	0.07 ± 6.86	0.947
LDL Cholesterol (mg/dl)	115.9 ± 33.4	106.9 ± 36.2	8.9 ± 27.8	0.042 *
Cholesterol: HDL ratio	4.2 ± 1.1	3.9 ± 1.1	0.27 ± 0.75	0.022 *
Triglycerides (mg/dl)	207.6 ± 121.7	189.8 ± 118.2	17.8 ± 82.9	0.167
BIA: Fat Mass (kg)	33.7 ± 13.3	31.9 ± 13.1	1.7 ± 3.1	0.001 *
BIA: Fat %	39.8 ± 9.3	38.3 ± 9.2	1.45 ± 2.35	<0.001 *
BIA: Fat-free Mass (kg)	48.2 ± 9.4	48.8 ± 9.4	0.57 ± 1.67	0.038 *
Weight (kg)	81.3 ± 18.3	80.9 ± 17.9	0.41 ± 2.59	0.317
Waist Circumference (cm)	100.4 ± 13.4	98.7 ± 13.2	1.66 ± 5.24	0.055
Hip Circumference (cm)	110.8 ± 15.6	110.6 ± 14.6	0.20 ± 3.36	0.695
Waist to Hip Ratio	0.91 ± 0.07	0.89 ± 0.06	0.01 ± 0.05	0.067
BMI (kg/m^2)	32.5 ± 7.1	32.0 ± 6.9	0.42 ± 1.04	0.012 *
DXA: Trunk Fat (kg)	16.2 ± 6.5	15.8 ± 6.3	0.40 ± 1.05	0.015 *
DXA: Trunk Lean (kg)	25.5 ± 4.8	25.4 ± 4.9	0.02 ± 1.04	0.885
DXA: Trunk Percent Fat (%)	37.4 ± 8.9	36.9 ± 8.7	0.52 ± 1.72	0.043 *
DXA: Total Fat (kg)	31.0 ± 12.4	30.4 ± 12.0	0.64 ± 1.61	0.013 *
DXA: Total Lean (kg)	49.2 ± 9.9	49.2 ± 10.2	0.05 ± 1.60	0.838
DXA: Total Percent Fat (%)	36.9 ± 9.0	36.4 ± 9.0	0.45 ± 1.19	0.018*

BIA = bioelectrical impedance analysis; BMI= body mass index; DXA = dual-energy X-ray absorptiometry; [a] This *p*-value is based on paired-samples *t*-test or Wilcoxon signed-ranks test comparing baseline to three-month changes; * *p*-value < 0.05; [b] Homeostasis Model Assessment of Insulin Resistance (HOMA-IR) = [fasting insulin (mU/L) × fasting glucose (mmol/L)] / 22.5.

Table S2. Results of linear regression analyses of baseline marker levels adjusted for the *Ala54Thr* polymorphism [a] on primary endpoint changes (Δ^b), ($n = 43$).

Linear Regression Model ($\hat{Y} = \alpha + \beta X_1 + \beta X_2 + \varepsilon_i$)	Baseline Marker	Baseline Marker *p*-value [c]	*Ala54Thr p*-Value
ΔFBG = α + βFBG_bl + βAla54Thr_group + ε_i	Fasting Blood Glucose (mg/dl)	<0.001 *	0.958
ΔHbA1c = α + βHbA1c_bl + βAla54Thr_group + ε_i	HbA1c (%)	<0.001 *	0.231
ΔInsulin = α + βInsulin_bl + βAla54Thr_group + ε_i	Insulin (uU/mL)	0.607	0.550
ΔHOMA-IR = α + βHOMA-IR_bl + βAla54Thr_group + ε_i	HOMA-IR	<0.001 *	0.513
ΔCholest. = α + βCholest_bl + βAla54Thr_group + ε_i	Total Cholesterol (mg/dl)	0.406	0.856
ΔHDL = α + βHDL_bl + βAla54Thr_group + ε_i	HDL Cholesterol (mg/dl)	0.070	0.421
ΔLDL = α + βLDL_bl + βAla54Thr_group + ε_i	LDL Cholesterol (mg/dl)	0.047 *	0.939
ΔChol:HDL = α + βChol:HDL_bl + βAla54Thr_group + ε_i	Cholesterol: HDL ratio	0.064	0.509
ΔTrig. = α + βTrig_bl + βAla54Thr_group + ε_i	Triglycerides (mg/dl)	0.013 *	0.888
ΔBIAFM = α + βBIAFM_bl + βAla54Thr_group + ε_i	BIA: Fat Mass (kg)	0.297	0.589
ΔBIAFat% = α + βBIAFat%_bl + βAla54Thr_group + ε_i	BIA: Fat %	0.359	0.786
ΔBIAFFM = α + βBIAFFM_bl + βAla54Thr_group + ε_i	BIA: Fat Free Mass (kg)	0.635	0.899
ΔWt = α + βWt_bl + βAla54Thr_group + ε_i	Weight (kg)	0.147	0.946
ΔWC = α + βWC_bl + βAla54Thr_group + ε_i	Waist Circumference (cm)	0.138	0.829
ΔHC = α + βHC_bl + βAla54Thr_group + ε_i	Hip Circumference (cm)	0.007 *	0.416
ΔW2H = α + βW2H_bl + βAla54Thr_group + ε_i	Waist to Hip Ratio	<0.001 *	0.610
ΔBMI = α + βBMI_bl + βAla54Thr_group + ε_i	BMI (kg/m^2)	0.120	0.943
ΔDXATrunkFat = α + βDXATrunkFat_bl + βAla54Thr_group + ε_i	DXA: Trunk Fat (kg)	0.065	0.899
ΔDXATrunkLean = α + βDXATrunkLean_bl + βAla54Thr_group + ε_i	DXA: Trunk Lean (kg)	0.787	0.670
ΔDXATrunk%Fat = α + βDXATrunk%Fat_bl + βAla54Thr_group + ε_i	DXA: Trunk Percent Fat (%)	0.145	0.354
ΔDXATotalFat = α + βDXATotalFat_bl + βAla54Thr_group + ε_i	DXA: Total Fat (kg)	0.077	0.953
ΔDXATotalLean = α + βDXATotalLean_bl + βAla54Thr_group + ε_i	DXA: Total Lean (kg)	0.290	0.716
ΔDXATotal%Fat = α + βDXATotal%Fat_bl + βAla54Thr_group + ε_i	DXA: Total Percent Fat (%)	0.622	0.581

[a] Ala/Ala (Ala54 homozygotes, G/G genotype) *vs.* Ala/Thr and Thr/Thr (Thr54 allele carriers, G/A and A/A genotypes combined); [b] Δ: Three-month -Baseline Change; [c] This *p*-value is based on the effect of the baseline marker as a predictor of the dependent change variable using the linear regression model shown; * *p*-value < 0.05.
